# Biomechanical effects of medial meniscus radial tears on the knee joint during gait: A concurrent finite element musculoskeletal framework investigation

**DOI:** 10.3389/fbioe.2022.957435

**Published:** 2022-10-10

**Authors:** Sentong Wang, Kazunori Hase, Shunsuke Kita, Shinya Ogaya

**Affiliations:** ^1^ Human-Mechanical System Laboratory, Graduate School of Systems Design, Tokyo Metropolitan University, Hachioji, Japan; ^2^ Human-Mechanical System Laboratory, Faculty of Systems Design, Tokyo Metropolitan University, Hachioji, Japan; ^3^ Biomechanics of Exercise and Sports in Physical Therapy Laboratory, Graduate Course of Health and Social Services, Saitama Prefectural University, Koshigaya, Japan; ^4^ Department of Rehabilitation, Soka Orthopedics Internal Medicine, Saitama, Japan; ^5^ Biomechanics of Exercise and Sports in Physical Therapy Laboratory, Department of Physical Therapy, Saitama Prefectural University, Koshigaya, Japan

**Keywords:** meniscus tear, finite element, musculoskeletal model, gait, knee, osteoarthritis

## Abstract

The biomechanical variation in the knee during walking that accompanies medial meniscal radial tears stemming from knee osteoarthritis (OA) has not been explored. This study introduced a finite element musculoskeletal model using concurrent lower limb musculoskeletal dynamics and knee joint finite element analysis in a single framework and expanded the models to include knees with medial meniscal radial tears and total medial meniscectomy. The radial tears involved three locations: anterior horn, midbody, and posterior horn with grades of 33%, 50%, and 83% of the meniscus width. The shear and hoop stresses of the tear meniscus and tibial cartilage contact load, accompanying tears, and postmeniscectomy were evaluated during the stance phase of the gait cycle using the models. In the 83% width midbody tear group, shear stress at the end of the tear was significantly greater than in the intact meniscus and other tear groups, and the maximum shear stress was increased by 310% compared to the intact meniscus. A medial meniscus radial tear has a much smaller effect on the tibial cartilage load (even though in the 83% width tear, the cartilage/total load ratio increased by only 9%). However, the contact force on the tibial cartilage with total postmeniscectomy was increased by 178.93% compared with a healthy intact meniscus, and the peak contact pressure after meniscectomy increased from 11.94 to 12.45 MPa to 17.64 and 13.76 MPa, at the maximum weight acceptance and push-off, respectively. Our study shows that radial tears with larger medial meniscus widths are prone to high stress concentrations at the end of the tears, leading to the potential risk of complete meniscal rupture. Furthermore, although the tears did not change the cartilage load distribution, they disrupted the circumferential stress-transmitting function of the meniscus, thus greatly increasing the likelihood of the onset of knee OA. The significant increase in the tibial cartilage load with total postmeniscectomy indicates a potential risk of OA flare-ups. This study contributes to a better understanding of meniscal tear-induced OA biomechanical changes during human activities and offers some potential directions for surgical guidance of meniscectomies and the prophylaxis and treatment of OA.

## Introduction

A meniscus tear is the most common damage associated with the meniscus, and it more commonly occurs on the medial compartment than on the lateral compartment (Fox et al., 2012). Moreover, radial tears are not uncommon in young patients (<50 years), often caused from trauma or degenerative processes (Terzidis et al., 2006; Howell et al., 2014), and can occur anywhere on either meniscus, anterior horn, posterior horn, or midbody (Fox et al., 2012). A meniscus tear can significantly affect the functions of the meniscus, such as shock absorption and load transmission, resulting in abnormal kinematics and redistribution of load on the knee (Kurosawa et al., 1980; Fox et al., 2015). The occurrence of these abnormal kinematics and mechanics often leads to increased cartilage wear or knee osteoarthritis (OA) changes (Felson, 2013). A large proportion of radial tears of the meniscus are considered irreparable, because they occur mostly in the white zone that lacks a blood supply (Scotti et al., 2013), so meniscectomy is often used to relieve pain and instability in the knee (Rao et al., 2015). However, meniscectomies have been reported to induce an increased risk of the progress of knee OA (Roemer et al., 2016). Hence, the biomechanical impact of meniscus radial tears and resultant meniscectomies on the knee joint needs to be investigated. Because activity-related radial tears are the most common (Terzidis et al., 2006), it is crucial to study the effects of radial tears on the kinematics and mechanics of the knee during human activities such as walking.

Previously, some researchers have used implantable pressure-sensitive film to study the contact mechanics between the torn menisci and articular cartilages in cadaveric knees in a knee machine that simulates walking (Bedi et al., 2010; Bedi et al., 2012; Gilbert et al., 2014). These works aimed to analyze the mechanical effect of the meniscus tears on the knee joint during human activities using an *in vitro* knee simulating apparatus. However, the *in vivo* knee physiological motion and loading pattern during activities were hard to replicate, and the contraction activity of muscles that act as “motors” for joint motion cannot be reproduced in cadavers. Moreover, the use of these sensors can severely alter meniscus translation and deformation, resulting in errors in predictions of the joint kinematics and contact mechanics (Hu et al., 2019). Thus, the kinematics and mechanics effects of meniscus tears on the knee during human activity are difficult to study using an *in vitro* approach.

Currently, a computational approach, the finite element (FE) method, has been proven to provide detailed geometric representations of torn menisci and can explain the stress alterations of menisci and load redistribution on the knee that accompanies meniscus tears (Kedgley et al., 2019; Li et al., 2019; Zhang et al., 2019). However, these studies cannot investigate the effect of meniscus tears on the knee during natural human activities, such as walking, because knee biomechanics were predicted based on simplified assumed loads and boundary conditions, such as fixed compressive load and flexion angles (Kedgley et al., 2019; Li et al., 2019; Zhang et al., 2019). In addition, muscles that produce the force that causes joint movement were not considered in biomechanical analysis of the knee (Mononen et al., 2013). Therefore, the biomechanical effect of the meniscus tears in the actual physiological conditions on the knee during human activities is still unclear.

This study aimed to elucidate 1) the stress alterations on the meniscus resulting from radial tears of the medial meniscus and 2) the effect of the tears and total medial meniscectomy on the knee biomechanics during the stance phase of the gait cycle. In the above discussion, we pointed out the problems of previous gait analyses using FE models: the inability to maintain a realistic representation of the knee joint tissue deformation and motion under natural physiological conditions. Therefore, we applied our previously developed finite element musculoskeletal (FEMS) model using a single concurrent framework combining the entire lower limb musculoskeletal dynamics and knee joint FE analysis, which has been proven to describe natural knee biomechanics during the gait (Wang et al., 2021; Wang et al., 2022), to clarify the biomechanical effect of tears on the knee during gait.

## Methods

### Subject experiments

A healthy male participant (age 29 years, height 175 cm, weight 80 kg) participated in the gait measurement. The participant was thoroughly informed about the purpose, methods, and caveats of the experiment. The experiment was approved by the research ethics committee of the Tokyo Metropolitan University. A straight, approximately 10-m-long walkway was prepared for the gait experiment. The participant was verbally instructed to “walk at a comfortable pace” with his preferred gait during the experiment. The marker-based motion trajectories (100-Hz sampling frequency, Vicon Nexus, Oxford Metrics Ltd., Oxford, United Kingdom) and ground reaction force (1000-Hz sampling frequency, TF-4060-D force plate, Tec Gihan Co., Ltd., Kyoto, Japan) were synchronously collected during a single stance phase.

### Finite element musculoskeletal model

**FIGURE 1:**
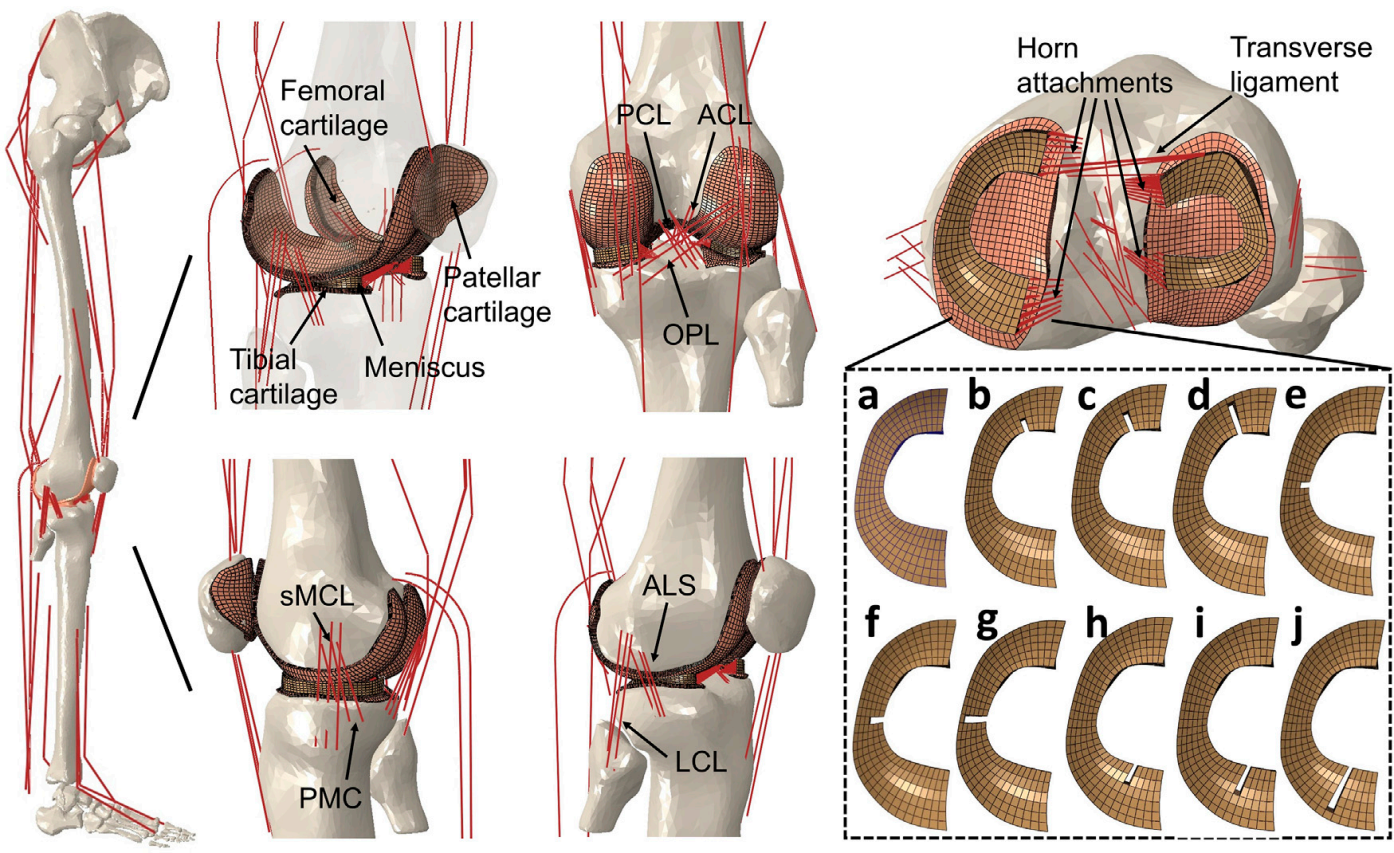
Right lower limb finite element musculoskeletal models containing knees with an intact healthy meniscus **(A)**; a medial meniscus with radial tears in the anterior horn involving three grades with widths of **(B)** 33%, **(C)** 50%, and **(D)** 83%; midbody with widths of **(E)** 33%, **(F)** 50%, and **(G)** 83%; and posterior horns with widths of **(H)** 33%, **(I)** 50%, and **(J)** 83%. A model without medial meniscus, equivalent to total meniscectomy, was also prepared (not shown). The lower limb finite element musculoskeletal model includes a 12-DOF knee joint, along with 20 muscles, ligaments (ACL, anterior cruciate ligament; PCL, posterior cruciate ligament; MCL, medial collateral ligament; PMC, posteromedial capsule; LCL, lateral collateral ligament; ALS, anterolateral structure; OPL, oblique popliteal ligament; transverse ligament), cartilage, the meniscus, and meniscus horn attachments.

**FIGURE 2:**
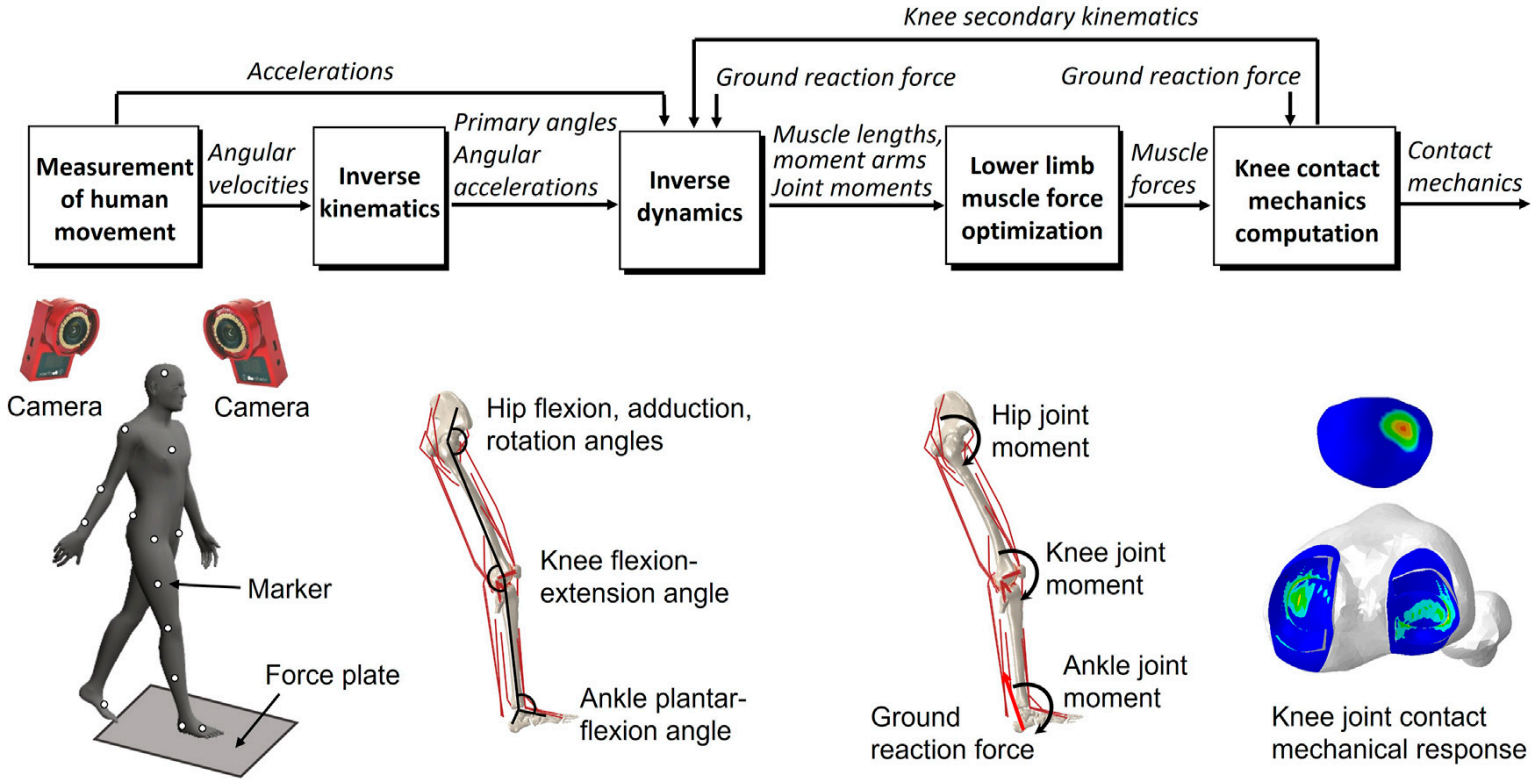
Overview of the dynamic gait simulation. Marker positions were input into the inverse kinematics to predict the joint primary angles (lumbar flexion–extension, hip joint 3-DOF rotations, knee joint flexion–extension, and ankle dorsiflexion–plantarflexion). The joint primary angles on each joint and ground reaction force measured from the force plate were input into the inverse dynamics to predict the muscle lengths, muscle moment arms, and right lower limb joint moments. The static optimization of the muscle force was performed with the joint moments, the muscle lengths, and the muscle moment arms as inputs to predict the muscle force. The knee contact mechanics were computed from the muscle forces and the ground reaction force to predict the knee joint secondary kinematics and contact mechanics. The predicted knee secondary kinematics were fed back to a new inverse dynamics analysis, and the muscle force optimization and knee contact mechanics were again computed.

A FEMS model of the right lower limb, including a healthy knee with FE analysis previously developed in ABAQUS/Explicit (SIMULIA, Providence, RI, United States) (Figure 1), was used in the study. The modeling approach of the FEMS framework (Figure 2) of the lower limb has been discussed and validated previously (Wang et al., 2021; Wang et al., 2022) but is summarized below. The FEMS model included the geometry of all right lower limb bones and the articular cartilages and meniscus of the knee, which were segmented manually and reconstructed from computed tomography and magnetic resonance imaging scans (Figure 1). The femoral, tibial, and patellar bones were meshed using rigid triangular shell elements, and the cartilage and meniscus were defined using elastic eight-node hexahedral elements. A spherical joint with three degrees of freedom (DOF) was used to represent the hip, joints with six DOFs represented the tibiofemoral and patellofemoral joints, and a hinge joint at the ankle with one DOF were included in the FEMS model of the right lower limb, as shown in Figure 1. The anterior cruciate ligament, posterior cruciate ligament, medial collateral ligament, posteromedial capsule, lateral collateral ligament, anterolateral structure, and oblique popliteal ligament cross tibiofemoral joint were modeled as nonlinear spring bundles with a force-strain relationship (Abdel-Rahman and Hefzy, 1998):

fi={0εi≤0ki 1(li−li 0)2 0<εi≤2ε l ki 2[li−(1+ε l)li 0]2ε l <εi (1)

where $f_{i}$ is the force sustained by the i th ligament, $l_{i}$ is the current length of the i th ligament, li 0 is the slack length of the i th ligament, ε l is the strain assumed to be constant at 0.03, and ki 1 and ki 2 are the stiffness coefficients of the spring elements representing the i th ligament for the nonlinear and the linear regions, respectively. The values of the material properties of the nonlinear spring elements are listed in Table 1 (Abdel-Rahman and Hefzy, 1998; Yu et al., 2001).

**TABLE 1:** Stiffness parameters of ligaments.

Ligament	Ligament bundle	Number of bundles	k 1 ( $N\;{mm}^{-2}$ )	k 2 ( $N\;{mm}^{-1}$ )
ACL	anterior	2	22.48	83.15
	posterior	2	26.27	83.15
PCL	anterior	2	31.26	125.00
	posterior	2	19.29	60.00
MCL	anterior	1	10.00	91.25
	oblique	1	5.00	27.86
	deep	1	5.00	21.07
PMC		3	12.00	52.59
LCL		3	10.00	72.22
ALS		3	5.00	19.00
OPL		3	3.00	21.42

The FEMS model included 20 representative one-dimensional linear element muscles on the lower limb: the gluteus maximus (3 units), iliopsoas, pectineus, rectus femoris, vastus medialis, vastus intermedius, vastus lateralis, semimembranosus, semitendinosus, biceps femoris (short and long heads), gastrocnemius (medial and lateral heads), soleus, tibialis (anterior and posterior), extensor halluces longus, and extensor digitorum longus. The wrapping between the muscles and femoral and tibial bone was considered in the model to represent the muscle paths (Ali et al., 2016). The mechanical properties of the muscles were represented using a Hill-type dynamic model containing a contractile element (active fiber force-length property) in parallel with a passive elastic element (passive fiber force-length property) (Zajac, 1989).

### Modeling of the meniscus with radial tears and meniscectomy

Modeling with FEMS, including knees with radial tears of the medial meniscus in the anterior horn, posterior horn, and midbody segment and without the medial meniscus (describing total meniscectomy), was an extension of the previously developed FEMS model with intact menisci. The medial meniscal model had radial tears in the anterior horn, midbody, and posterior horn involving three grades: 1) one-third width (33%), 2) one-half width (50%), and 3) five-sixths width (83%). These were created by partially removing the mesh from an intact medial meniscus (Figure 1). The intact and torn menisci were defined to be transversely isotropic with radial and axial moduli of 20 MPa and a circumferential modulus of 140 MPa (Donahue et al., 2002). The in-plane and out-of-plane Poisson’s ratios were 0.2 and 0.3, respectively (Donahue et al., 2002; Yao et al., 2006). The articular cartilage was considered as a linear elastic isotropic material with an elastic modulus of 5 MPa and a Poisson’s ratio of 0.46 (Li et al., 2001). It should be noted that to exclude the biomechanical effect of biological changes of tissue properties in the knee, only changes in the geometry of the meniscus tears were considered; in other words, the material property changes of tissues after tearing were not considered. The contact behaviors were defined with a coefficient of friction of 0.04 (McCann et al., 2009) for all contact pairs in the intact knee and knees with meniscus tear models: the femoral cartilage–medial and lateral meniscus, femoral cartilage–medial and lateral tibial cartilages, femoral cartilage–patellar cartilage, and between the meniscus and tibial cartilages on the medial and lateral sides. In the total meniscectomy model, the medial meniscus was resected, and the contact behavior of cartilage and the medial meniscus was not included.

The anterior transverse ligament connecting the anterior convex margin of the lateral meniscus to the anterior end of the medial meniscus was modeled as multiple linear spring elements with a stiffness of 12.5 N/mm (Donahue et al., 2003). The four meniscal horn attachments were assumed as multiple linear spring elements firmly connecting the meniscal horn faces and the tibial bone (Figure 1). The spring constants were calculated from Young’s modulus reported for the horn attachments (Hauch et al., 2009):

$$k_{hp}=\frac{E}{n_{h}l_{hp}}a_{h}$$ (2)

where h indicates the meniscal horn; p indicates the linear spring, where $k_{hp}$ is the p th spring stiffness for the h th meniscal horn; $l_{hp}$ is the p th spring length of the h th meniscal horn; E is Young’s modulus of the meniscal horn; $a_{h}$ is the h th horn face area; and $n_{h}$ is the number of spring elements for the h th meniscal horn. The spring length of each spring element was calculated from the insertions of the spring at the node of the horn face and the node of the tibial attachments.

### Concurrent finite element musculoskeletal framework for gait analysis

The gait analysis considering lower limb motion achieved through inverse kinematics, inverse dynamics, muscle force optimization, and FE analysis on the knee, using inertial measurement unit sensors or motion capture system has been described previously (Wang et al., 2021; Wang et al., 2021; Wang et al., 2022) but is briefly presented below. In the study, considering that measurement accuracy was more important than measurement convenience, the gait analysis was performed using a motion capture system and force plate, generally regarded as the gold standard for motion analysis, instead of inertial measurement sensor units. An inverse kinematics analysis was used to derive the primary angles (hip joint flexion–extension, internal–external rotation, and abduction–adduction angles; knee joint flexion-extension angle; and ankle joint dorsiflexion–plantarflexion angle) of joints from marker trajectories measured in a human gait experiment. These primary joint angles and the measured ground reaction force were input to the inverse dynamics analysis to determine the joint moments, muscle lengths, and muscle moment arms. The muscle lengths and muscle moment arms were input to a static optimization algorithm to estimate the muscle forces by satisfying an equilibrium equation of the joint moment, and the muscle forces were optimized by minimizing the sum of the cube of the muscular activation $s_{m}$ as follows (Hase and Yamazaki, 2002):

$$M_{j}\left(\theta_{j},e_{s}\right)=\underset{m}{\sum }\left[r_{jm}\left(\theta_{j},e_{s}\right)\times f_{jm}\left(s_{m}\right)\right]$$ (3)

$$C=\min\underset{m}{\sum }s_{m}^{3}$$ (4)

where j indicates the joint, m indicates the muscle, s indicates the knee secondary kinematics, $M_{j}\left(\theta_{j},e_{s}\right)$ is the moment of the j th joint, $\theta_{j}$ is the primary angle of the j th joint, $e_{s}$ is the secondary kinematics of the knee joint (note that the hip and ankle joints only include primary angles) for the s th degree of freedom, and $r_{jm}\left(\theta_{j},e_{s}\right)$ is the moment arm vector for the j th joint. With each muscular activation considered as a variable, the force of the m th muscle can be expressed as a function $f_{jm}\left(s_{m}\right)$ of the activation about the j th joint. In addition, the muscular activations were constrained to be in the range of zero to one (inclusive).

The static optimization method was performed by implementing subroutines written in MATLAB (R2018b, MathWorks, MA, United States) using the interior-point method, which combines concurrent FE contact analysis on the knee joint in ABAQUS/Explicit at 16 evenly distributed time points (0%, 6.67%, 13.33%, 20%, 26.67%, 33.33%, 40%, 46.67%, 53.33%, 60%, 66.67%, 73.33%, 80%, 86.67%, 93.33%, and 100% of the stance phase) during one stance phase. The secondary kinematics of the knee, which were determined entirely from the interaction of the joint contact mechanics, muscle forces, and ligament restraint, were fed back to a new inverse dynamics analysis updating muscle lengths and muscle moment arms until the stability of optimization was ensured with the convergence criteria for equilibrium in the joint moment. The FEMS models containing an intact knee joint, damaged knee joints with meniscal tears, and a joint without a meniscus were used to perform the optimization procedures, separately, under the same input condition (marker position and ground reaction force).

## Results

**FIGURE 3:**
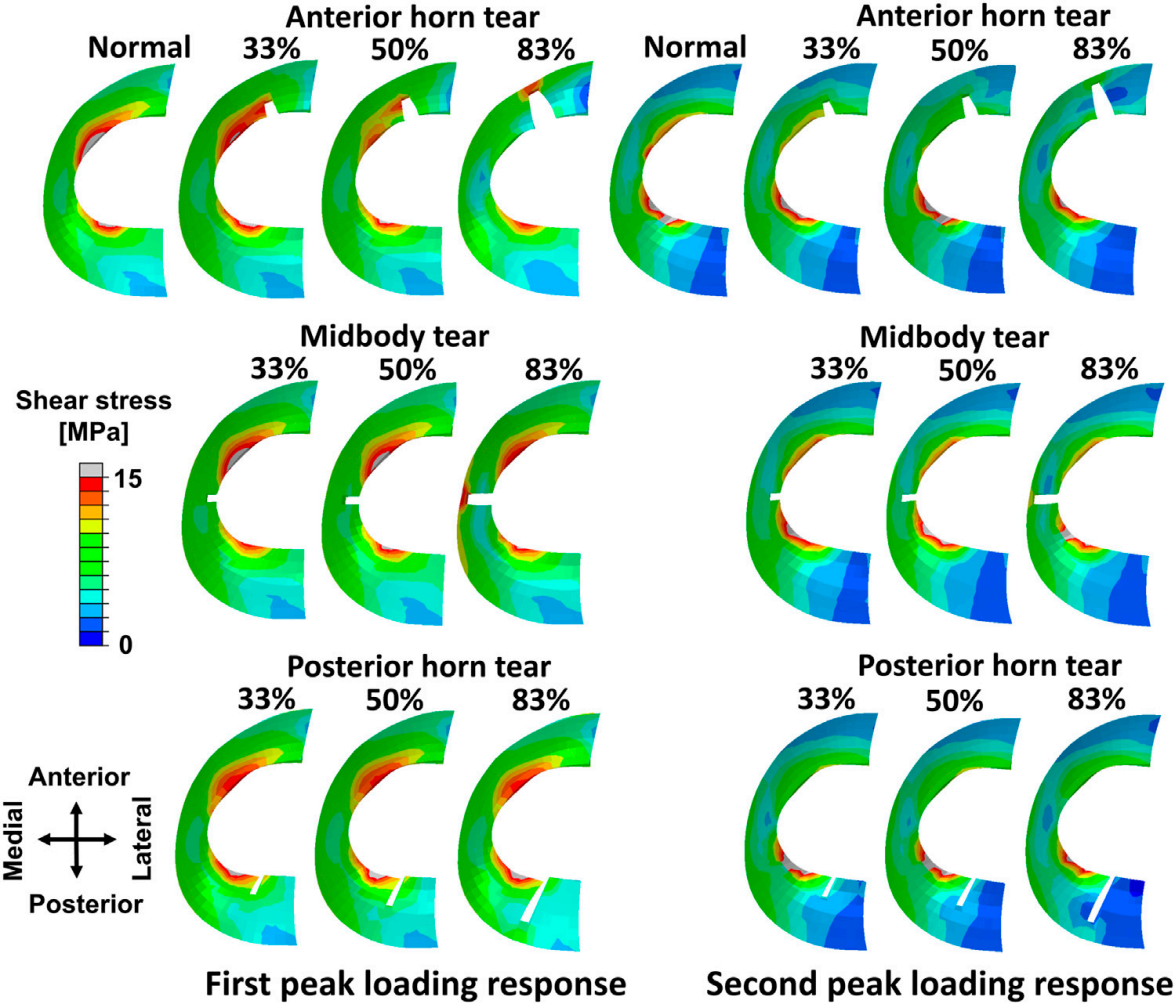
Results of shear stress distribution on the medial meniscus involving a healthy knee and a knee with radial tears in the medial meniscus during the maximum weight acceptance and push-off.

The peak shear stress, occurred on the white zone of the medial meniscus in the intact meniscus during the maximum weight acceptance of the stance phase (1st peak of the vertical ground reaction force) (Figure 3). The shear stress distribution of the meniscus changed as the anterior horn tear widened, shear stress in the anterior horn tear increased significantly, and the peak shear stress occurred at the end (deepest portion of tear near the side of the red zone) of the 83% tear. The changes in shear stress distribution that occurred on the medial meniscus with the midbody tear were not observed at widths of 33% and 50%. Tears of the posterior horn did not alter the shear stress distribution on the meniscus during the maximum weight acceptance. During the push-off at the stance phase (2nd peak of the vertical ground reaction force), changes in the shear stress on the medial meniscus were barely observed at three locations with tears.

**FIGURE 4:**
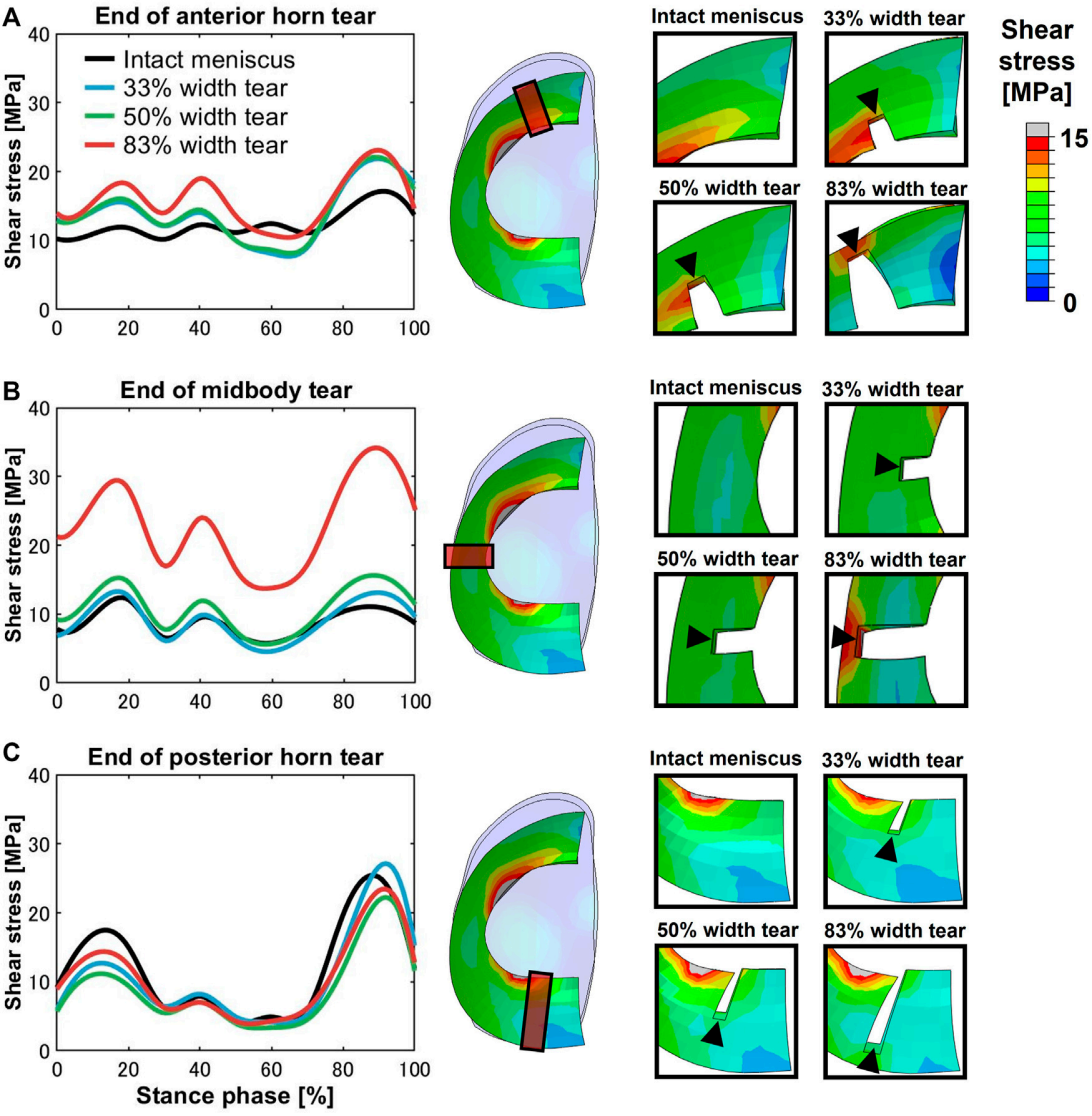
Shear stress differences on an intact meniscus and a meniscus with a medial radial tear at the ends of the tears. The left subfigures show the magnitude of stress at the **(A)** anterior horn, **(B)** midbody, and **(C)** posterior horn, involving an intact meniscus and 33%, 50%, and 83% width tears during the stance phase of the gait cycle. The right subfigures indicated the tear locations (orange box) at the anterior horn, midbody, and posterior horn and enlarged views of the tear locations.

High shear stress was observed at the ends of meniscal anterior horn tears, including all three grades compared to the intact meniscus, except during the terminal stance (50%–70% stance phase) (Figure 4A). In the 83% midbody tear group, the shear stress located at the end of the tear was significantly more prominent than that in the intact meniscus and other tear groups, and the maximum shear stress at the 90% stance phase was increased by 310% compared to the intact meniscus (Figure 4B). Alterations in the ends of the meniscal posterior horn tear groups were not detected during the stance phase (Figure 4C).

**FIGURE 5:**
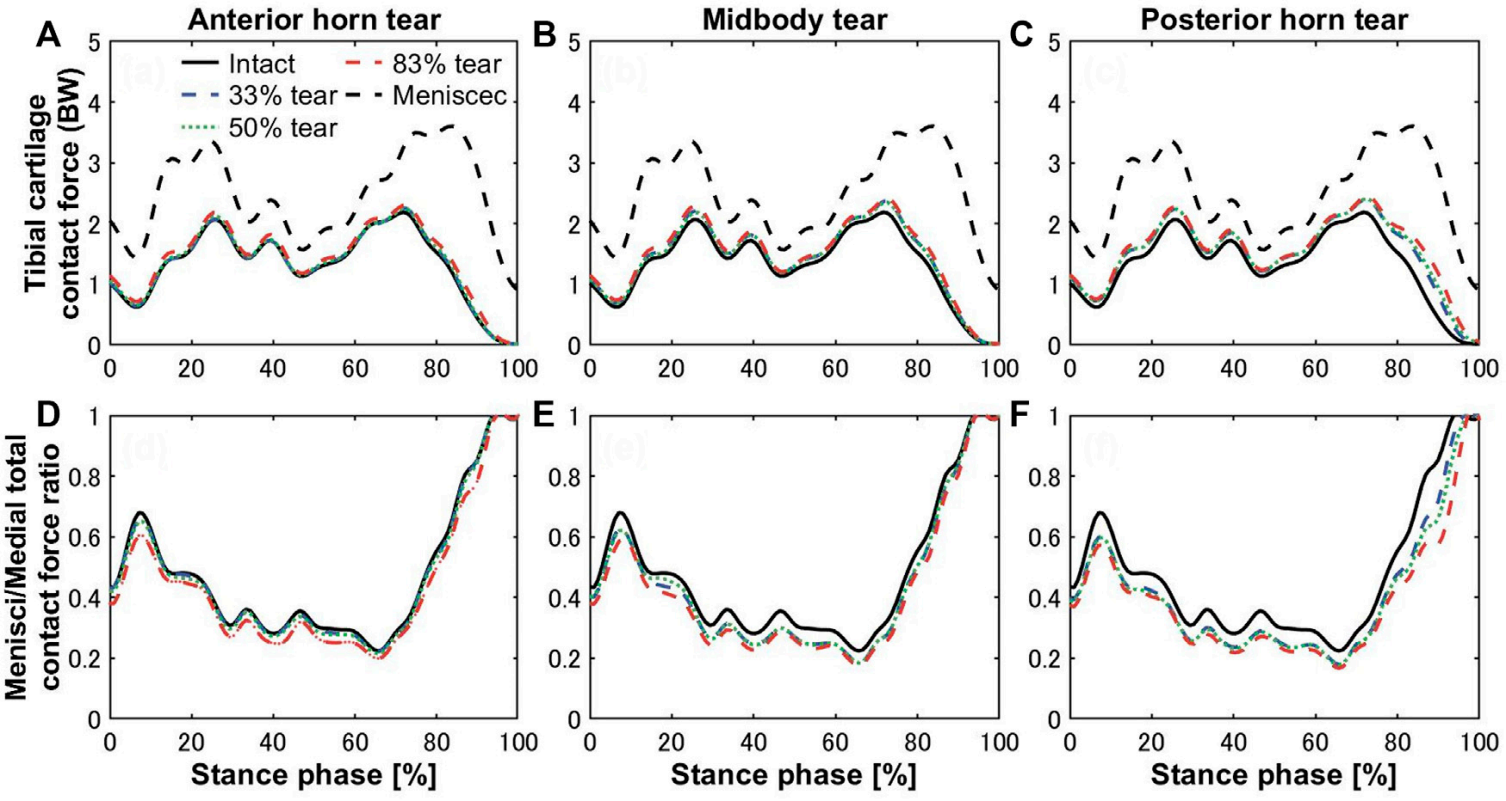
Contact forces on medial tibial cartilages of the intact knee (black solid curves) compared with total meniscectomy (black dashed curves) and **(A)** anterior horn, **(B)** midbody, and **(C)** posterior horn tears with widths of 33% (blue dashed curve), 50% (green dotted curve), and 83% (red dashed curve) during the stance phase of the gait cycle. The lower panels show the ratios of the medial meniscal contact forces to total knee joint contact forces on the medial side with tears in the **(D)** anterior horn, **(E)** midbody, and **(F)** posterior horn.

**FIGURE 6:**
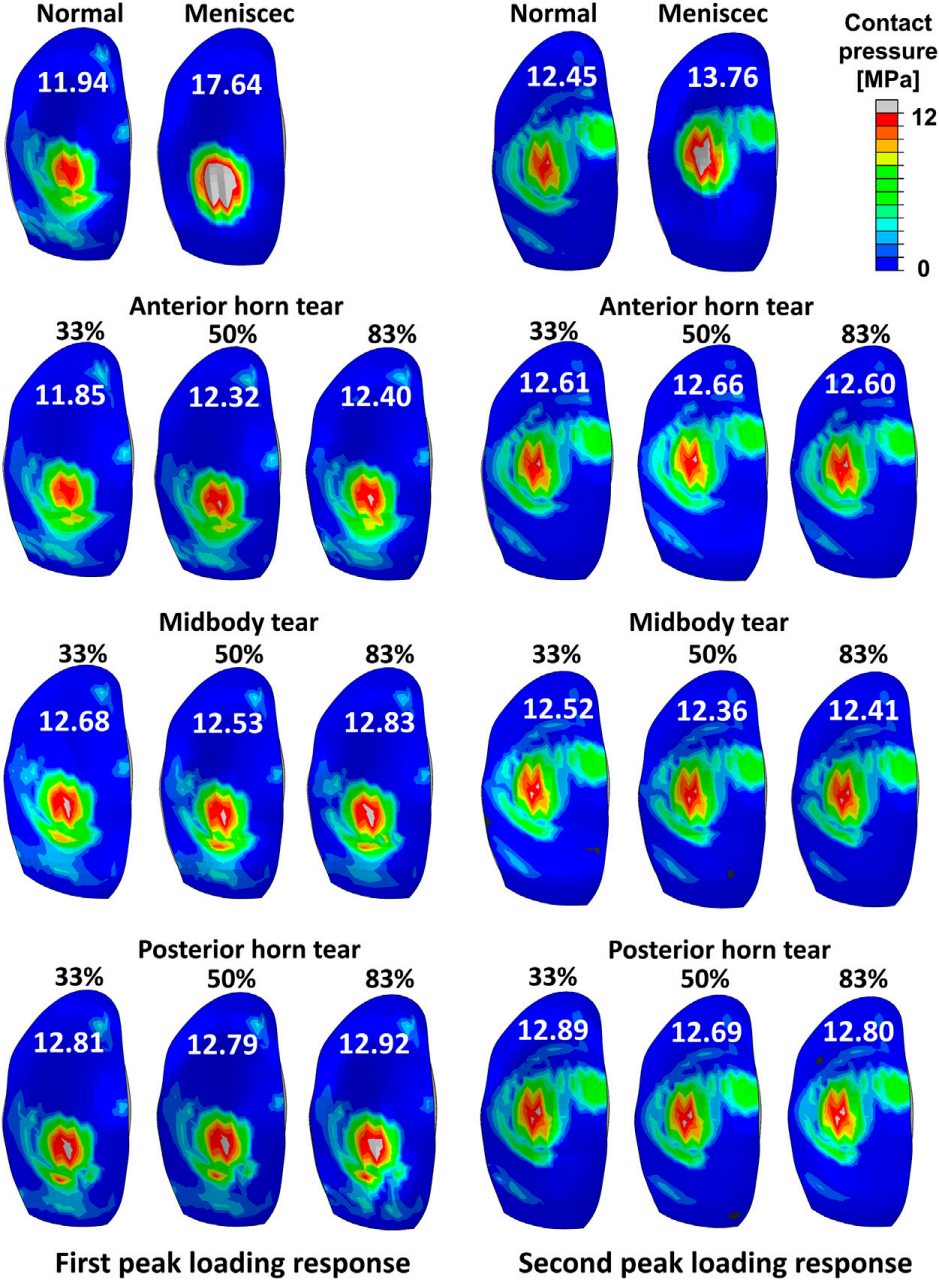
Results of the contact pressure distribution and resultant peak values (white words) occurring on the medial tibial cartilage obtained by a healthy intact knee, total meniscectomy knee, and radial tears during the maximum weight acceptance and push-off.

The tibial cartilage contact forces were hardly affected by radial tears in the medial meniscus (Figures 5A–C). The ratio of the medial meniscus contact force to the whole medial contact force was 0.61 on average in the intact meniscus; that is, the tibial cartilage shared 39% of the total medial load, which illustrates the essential function of the medial meniscus in load sharing. An insignificant increase in the load shared by the tibial cartilage to the total medial side was observed with tears (Figures 5E,F), consistent with previous experimental *in vitro* results (Walker et al., 2015). However, after meniscectomy, the maximum contact force on the medial tibial cartilage was 3.6 times the bodyweight, compared to 2.17 times in the intact healthy knee. The contact pressure distributions on the medial tibial cartilages in the intact, torn, and meniscectomy knees are shown in Figure 6 at the maximum weight acceptance and push-off. The peak contact pressure on the tibial cartilage increased after meniscectomy to 17.64 MPa and 13.76 MPa, compared with the healthy knee (11.94 MPa and 12.45 MPa) at the maximum weight acceptance and push-off, respectively.

## Discussion

In this study, models of the lower limb with a medial meniscus having radial tears and without a medial meniscus were developed based on a previously developed FEMS model (Wang et al., 2021; Wang et al., 2022), which has been proven to describe realistic knee biomechanics during the gait cycle. The predicted knee biomechanics results of the model containing an intact knee were compared with experimental *in vivo* and *in vitro* results (Kozanek et al., 2009; Bergmann et al., 2014; Gilbert et al., 2014; Clément et al., 2018) and proven to be reliable. We evaluated the knee biomechanical changes after meniscus tears and total meniscectomy using the models.

**FIGURE 7:**
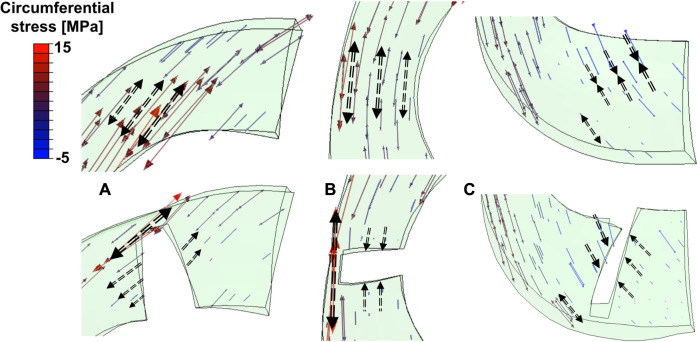
Tensor plots of circumferential stress for the **(A)** anterior horn, **(B)** midbody, and **(C)** posterior horn with 83% width tears. The color changes from blue to red represent the stress variation from small (negative, compressive stress) to large (positive, tensile stress) on the tensor plot. Dashed arrows indicated circumferential stress in tensile or compressive directions. Thicker longer arrows represent higher magnitudes.

The anterior horn with intact meniscus bore stable shear stress with an average value of 12.27 MPa (Figure 4A) from the knee during the stance phase of the gait, compared with 6.72 MPa (Zhang et al., 2019) and 15.34 MPa (Zhang et al., 2019) predicted by other finite element simulation during slight flexion. However, some investigators also reported smaller results of less than 4 MPa (Li et al., 2019; Li et al., 2020); these differences might be due to the definition of the geometry, material properties, and contact behavior of the knee joint. In the medial meniscus, the mobility of the anterior horn was most prominent (Thompson et al., 1991; Vedi et al., 1999), and there was basically no obvious fluctuation in the shear stress of the anterior horn in the intact knee during the stance phase (Figure 4A), which may suggest that the anterior horn plays a role in stabilizing load transmission, but only during knee extension. As shown in Figure 7A, circumferential tensile stress occurred in the anterior horn of the intact meniscus, which showed a uniform decreasing trend from the inner circumference to the outer circumference. The shear stresses that accompanied the tear increased significantly at the ends of the anterior horn tears (Figure 4A); this was due to the destruction of the circumferential collagen fibers on the inner circumference, which results in the transfer of the load to the tear and produces more significant circumferential stress (Figure 7A). When the 83% width tear occurs, the changing trend of the circumferential tensile stress from the inner to the outer circumference changes from a uniform decrease to a gradual increase. Especially, the stress at the end of the tear increases extremely, and all the tear areas were stretched, which may lead to further tearing or complete rupture. On the intact meniscus, the average shear stress experienced by the midbody was 8.77 MPa, which is less than 12.27 MPa for the anterior horn and 11.54 MPa for the posterior horn. The anterior and posterior horns carry the anteroposterior shear forces applied to the femur, while the midbody primarily provides stability against medial subluxation by the midbody of the meniscus through hoop stress transmission (Hwang et al., 2012). The midbody of the intact meniscus exhibited a uniform hoop tensile stress trend that increases from the inner circumference to the outer circumference, as shown in Figure 7B. Therefore, the higher hoop stress from the outer circumference can make the meniscus firmly lock onto the medial femoral condyle (Figure 7A). The tears with widths of 33% and 50% hardly affected changes in the midbody shear stress distribution (Figures 3, 4B). However, substantial stress concentrations were detected at the end of the 83% midbody tear (Figure 4B), which may mean that longer tears at the midbody severely disrupt more circumferential fibers and hoop stress transmission, causing an increase in stress. Stresses reached the experimentally measured failure stress range of 20–170 MPa (Kohn et al., 1992). We observed that the hoop tensile stress in the tear region was replaced by compressive stress as the tear increased, resulting in the need for extremely high tensile stress at the tear to maintain circumferential stress transmission, as shown in Figure 7B. The observed compressive stress was favorable for compressing the tear surfaces together, resulting in a stable tear and possibly more favorable healing conditions. However, a study reported that five of the six radial tears had no evidence of healing and one had become longer (Weiss et al., 1989). Henning et al. (1988) suggested that only shallow radial tears do not require treatment because they heal spontaneously or remain asymptomatic. Our observations contradict these conclusions from clinical reports. This may be because the defined isotropic material models cannot accurately calculate the stresses within a fibrillar tissue such as the meniscus, and a fibril-reinforced material model, which can represent fluid pressure and site-specific collagen fiber orientation, should be used for accurate simulations (Klets et al., 2016). Even so, this finding could indicate that alongside vascularity, biomechanics may play an essential role in healing for nonoperative treatment or postoperative meniscal repair. The translation is significant in the anterior horn and small in the posterior horn of the medial meniscus. Therefore, stress readily accumulated at the junction of the middle and the posterior portions of the medial meniscus (Figure 3), consistent with the results reported in other studies (Bin et al., 2004). The posterior horn of the intact meniscus carried a high percentage of the load overall, with shear stress reaching a peak of 25.16 MPa at higher flexion angles during preswing, compared with 17.08 MPa for the anterior horn and 10.98 for the midbody during the same gait phase. The low translation of the posterior horn of the medial meniscus is a potential mechanism for meniscal tears, with a resultant “trapping” of the fibrocartilage between the femoral condyle and the tibial plateau during high flexion (Fox et al., 2012), possibly contributing to the high circumferential compressive stress distribution. As shown in Figure 7C, the tears did not change the hoop compressive stress distribution, and the stress that occurred at the tear surfaces caused them to be compressed together. Apart from vascularity, this may explain why radial tears in this region are most amenable to healing (Belzer and Cannon, 1993). In the addition, the slackness of the meniscus horn attachment also affects the loading of the meniscus horn. Guess et al. (2016) showed that increased meniscal extrusion occurs with laxity of the attachment, and increased laxity results in loss of meniscal function. In contrast, simulating an overly stiff meniscus horn attachment might lead to an overloading of the meniscal horns, and the study provides important guidance for related research on meniscus modeling.

Bergmann group has conducted measurements of the *in vivo* tibiofemoral joint contact loads, which are 2.61 body weight (Kutzner et al., 2010) and 3.98 body weight (Dreyer et al., 2022), respectively, using instrumented implants. These studies directly measured the knee joint load of multiple patients and performed statistical analysis. Although these measurement results are not natural knee data, and due to the material properties of the total knee arthroplasty prosthesis, prosthesis geometry, and interprosthesis contact, the represented behavior varies widely from natural or removed meniscal knees. However, they can be used as a quantitative reference to properly verify the accuracy of our prediction results. The peak contact forces of 4.42 BW (Navacchia et al., 2019) and 4.02 BW (Hume et al., 2019) during the gait cycle were predicted by the Shelburne group using a finite element knee model without a meniscus. As far as we know, the joint contact force is generally accepted as being between two and 4.5 BW (Kumar et al., 2013; Bergmann et al., 2014; Richards et al., 2018; Hume et al., 2019; Shu et al., 2022). In general, the results predicted by computational models are usually greater than those predicted by *in vivo* experiments (Kutzner et al., 2010; Esrafilian et al., 2020; Wang et al., 2022). The role of the meniscus in sharing knee joint loading on the medial side was most significant during the loading response (0%–20% stance phase) and preswing (80%–100% stance phase) (Figure 5E,F). The ratio of the medial meniscus contact force to the whole medial contact force was 0.57 and 0.78 on average on the intact meniscus during the loading response and preswing, respectively. The trends of increased cartilage load caused by tearing were not apparent, compared with the ratio of meniscus–medial total contact force on the intact meniscus, which increased by 2.6%, 5.1%, and 7.7% on average in the anterior horn, midbody, and posterior horn tear groups, respectively, during the stance phase. Of these, even though the presence of an 83% width tear of the posterior horn during the preswing resulted in the most significant increase in cartilage load, the cartilage/total load ratio increased by only 9%. The findings suggested that medial meniscus radial tears have lesser influence on the tibial cartilage loading (Figure 5), consistent with previous experimental results (Bedi et al., 2010; Bedi et al., 2012), however, some studies have also concluded that meniscal tears lead to increased cartilage loading (Zhang et al., 2015; Sukopp et al., 2021). Previously, investigators have pointed out that meniscal tears in a healthy knee may ultimately lead to the occurrence of knee OA due to the loss of much of the meniscal functionality, such as circumferential stress transmission (Englund et al., 2009). According to results from previous case-control studies, OA is more likely to occur in the knee with meniscus tears without arthropathy than in knees with intact menisci, suggesting that meniscal tears occur before any visible cartilage changes (Englund et al., 2009). The cartilage loads were not significantly increased by the meniscal tears, but OA may begin to occur as tears result in loss of most of the meniscus function, which requires extreme vigilance. Therefore, the evaluation of meniscal function in addition to mechanical evaluation of cartilage is critical.

The peak contact pressure was 12.45 MPa on a healthy knee in our model. The results might be acceptable, compared with other computational studies, which reported results of 7.9 (Park et al., 2019), 13.0 (Halonen et al., 2017), and 15 MPa (Kłodowski et al., 2016). Bedi et al. (2010) reported maximum contact pressures of 7.4 ± 0.6 MPa and 6.4 ± 1.1 MPa (Bedi et al., 2012) in vitro studies on a cadaver knee joint with film contact pressure sensors to simulate gait conditions. Our calculated maximum contact pressures were high compared to these *in vitro* experiment results. However, with *in vitro* experiments, the contraction activity of muscles cannot be reproduced in cadavers, which may have resulted in the measurement of relatively small contact loads. Moreover, the use of these sensors can severely alter meniscus translation and deformation, possibly resulting in prediction errors of the contact mechanics. The maximum peak contact pressure on the tibial cartilage increased to 17.64 MPa after meniscectomy, which indicated a risk of cartilage damage (Kempson et al., 1971). The contact force on the tibial cartilage after total meniscectomy was increased by 178.93% compared with the intact meniscus knee (Figure 5). The magnitude of the peak contact pressure on the tibial cartilage with total meniscectomy greatly increased, and the contact distribution was significantly altered compared to the intact and tear meniscus groups. Previous research reported that radial tears extending to the periphery result in a loss of hoop tension, which has been described as functionally equivalent to a total meniscectomy (Allaire et al., 2008). Therefore, total meniscectomy is the mainstream treatment for long radial tears. However, despite the benefits of short-term pain relief, meniscectomy resulted in a substantial cartilage load that may increase the risk of OA progression; in other words, the effect on the knee biomechanics can be much worse than the meniscus tear itself. Our finding demonstrated that although tears can disrupt the meniscal circumferential stress transfer function, even wider meniscal radial tears have little effect on cartilage loading, whereas meniscectomy has a significant effect on the cartilage load. Therefore, we emphasize the importance of meniscal preservation whenever feasible, including consideration of meniscus repair or the implantation of an artificial meniscus.

This study had some limitations. First, the FEMS lower limb model and gait data were not from the same subject. The most accurate computational analysis of knee mechanics requires subject-specific model geometry and human motion data. Second, only one subject participated in the gait measurement experiment. Due to individual differences, the generalizability of our findings may be affected by random individual traits. However, using the same walking pattern of the same subject as input data while only changing the meniscus tear model eliminates the influence of the walking pattern itself on changes in joint load; the effects of meniscal tears can be directly evaluated. Therefore, we decided to perform the analysis using the data of one trial with one subject without considering the walking pattern and the subject’s own gait variation. In addition, analysis using the same gait measurement data was also beneficial for eliminating the influence of different gait patterns themselves in meniscal tear patients on changes in joint load. Third, the ligaments were simplified as one-dimensional elastic elements to reduce the computational expense. However, because wrapping and realistic soft tissue contact are not included in the model, the effect of the medial collateral ligament and oblique popliteal ligament contacting the medial meniscus could not be represented, which may have implications for meniscal mechanical prediction. Fourth, to exclude the influence of mesh conditions, we did not reconstruct and remesh the geometries of the menisci with tears; instead, these were created by partially removing the mesh from an intact medial meniscus. However, this modelling was unable to replicate a more realistic meniscus with tears geometry and may lead in errors in predictions of the joint contact mechanics. In addition, the division of the mesh was based on experience in the study; however, to identify the optimum mesh size, a mesh convergence analysis should be conducted. A final limitation was that the articular cartilages were defined as linear elastic isotropic material, whereas a biphasic fibril-reinforced material might better approximate the representation of cartilage’s dynamic response (Brindle et al., 2001). However, studies have also shown that the properties of linear elastic materials can accurately mimic the overall behavior of cartilage (Bell et al., 2009).

## Conclusion

In this study, FEMS models of the lower limb, including knees with radial tears of the medial meniscus and total medial meniscectomy, were developed and used to investigate the effects of radial meniscal tears and total meniscectomy on the meniscal stress changes and biomechanical redistribution on the knee. Our research demonstrated that wider radial tears of the medial meniscus are prone to high-stress concentrations at the end of the tears, leading to the potential risk of developing complete meniscal ruptures. In addition, although the tears did not cause changes in cartilage load distribution, they disrupted the circumferential stress transmitting function of the meniscus; thus, knee OA may begin to develop. Significantly increased load on the tibial cartilage with the postmeniscectomy model indicated potential risks for the onset of OA. Therefore, surgical procedures such as meniscectomy should be applied conservatively; that is, excessive removal of meniscal tissue should be avoided. The modeling may provide a potential clinical tool for surgical decisions for patients with meniscus and other soft tissue injures, including more accurate pathology analysis and treatment of OA. Overall, the FEMS is expected to provide doctors with a reference for diagnosis.

## Data Availability

The original contributions presented in the study are included in the article/supplementary material, further inquiries can be directed to the corresponding authors.

## References

[B1] Abdel-Rahman E. M. Hefzy M. S. (1998). Three-dimensional dynamic behaviour of the human knee joint under impact loading. Med. Eng. Phys. 20, 276–290. 10.1016/S1350-4533(98)00010-1 9728679

[B2] Ali A. A. Shalhoub S. S. Cyr A. J. Fitzpatrick C. K. Maletsky L. P. Rullkoetter P. J. (2016). Validation of predicted patellofemoral mechanics in a finite element model of the healthy and cruciate-deficient knee. J. Biomech. 49, 302–309. 10.1016/j.jbiomech.2015.12.020 26742720 PMC4761469

[B3] Allaire R. Muriuki M. Gilbertson L. Harner C. D. (2008). Biomechanical consequences of a tear of the posterior root of the medial meniscus. Similar to total meniscectomy. J. Bone Jt. Surgery-American Volume 90, 1922–1931. 10.2106/JBJS.G.00748 18762653

[B4] Bedi A. Kelly N. H. Baad M. Fox A. J. S. Brophy R. H. Warren R. F. (2010). Dynamic contact mechanics of the medial meniscus as a function of radial tear, repair, and partial meniscectomy. J. Bone Jt. Surgery-American Volume 92, 1398–1408. 10.2106/JBJS.I.00539 20516315

[B5] Bedi A. Kelly N. H. Sc B. Baad M. Fox A. J. S. Sc M. (2012). Dynamic contact mechanics of radial tears of the lateral meniscus: Implications for treatment. Arthrosc. J. Arthrosc. Relat. Surg. 28, 372–381. 10.1016/j.arthro.2011.08.287 22074620

[B6] Bell J. S. Winlove C. P. Smith C. W. Dehghani H. (2009). Modeling the steady-state deformation of the solid phase of articular cartilage. Biomaterials 30, 6394–6401. 10.1016/j.biomaterials.2009.08.026 19716172

[B7] Belzer J. P. Cannon W. D. (1993). Meniscus tears: Treatment in the stable and unstable knee. J. Am. Acad. Orthop. Surg. 1, 41–47. 10.5435/00124635-199309000-00006 10675855

[B8] Bergmann G. Bender A. Graichen F. Dymke J. Rohlmann A. Trepczynski A. (2014). Standardized loads acting in knee implants. PLoS ONE 9, 86035. 10.1371/journal.pone.0086035 PMC3900456 24465856

[B9] Bin S. Kim J. Shin S. (2004). Radial tears of the posterior horn of the medial meniscus. Arthrosc. J. Arthrosc. Relat. Surg. 20, 373–378. 10.1016/j.arthro.2004.01.004 15067276

[B10] Brindle T. Nyland J. Johnson D. L. (2001). The meniscus: Review of basic principles with application to surgery and rehabilitation. J. Athl. Train. 36, 160–169. 16558666 PMC155528

[B11] Clément J. Toliopoulos P. Hagemeister N. Desmeules F. Fuentes A. Vendittoli P. A. (2018). Healthy 3D knee kinematics during gait: Differences between women and men, and correlation with X-ray alignment. Gait Posture 64, 198–204. 10.1016/j.gaitpost.2018.06.024 29933182

[B12] Donahue T. L. H. Hull M. L. Rashid M. M. Jacobs C. R. (2002). A finite element model of the human knee joint for the study of tibio-femoral contact. J. Biomech. Eng. 124, 273–280. 10.1115/1.1470171 12071261

[B13] Donahue T. L. H. Hull M. L. Rashid M. M. Jacobs C. R. (2003). How the stiffness of meniscal attachments and meniscal material properties affect tibio-femoral contact pressure computed using a validated finite element model of the human knee joint. J. Biomech. 36, 19–34. 10.1016/s0021-9290(02)00305-6 12485635

[B14] Dreyer M. J. Trepczynski A. Nasab S. H. H. Kutzner I. Schütz P. Weisse B. (2022). European Society of Biomechanics S.M. Perren Award 2022: Standardized tibio-femoral implant loads and kinematics. J. Biomech. 141, 111171. 10.1016/j.jbiomech.2022.111171 35803037

[B15] Englund M. Guermazi A. Lohmander S. L. (2009). The role of the meniscus in knee osteoarthritis: A cause or consequence? Radiol. Clin. North Am. 47, 703–712. 10.1016/j.rcl.2009.03.003 19631077

[B16] Esrafilian A. Stenroth L. Mononen M. E. Tanska P. Avela J. Korhonen R. K. (2020). EMG-assisted muscle force driven finite element model of the knee joint with fibril-reinforced poroelastic cartilages and menisci. Sci. Rep. 10, 3026. 10.1038/s41598-020-59602-2 32080233 PMC7033219

[B17] Felson D. T. (2013). Osteoarthritis as a disease of mechanics. Osteoarthr. Cartil. 21, 10–15. 10.1016/j.joca.2012.09.012 PMC3538894 23041436

[B18] Fox A. J. S. Bedi A. Rodeo S. A. (2012). The basic science of human knee menisci structure, composition, and function. Sports Health. 4, 340–351. 10.1177/1941738111429419 23016106 PMC3435920

[B19] Fox A. J. S. Wanivenhaus F. Burge A. J. Warren R. F. Rodeo S. A. (2015). The human meniscus: A review of anatomy, function, injury, and advances in treatment. Clin. Anat. 28, 269–287. 10.1002/ca.22456 25125315

[B20] Gilbert S. Chen T. Hutchinson I. D. Choi D. Voigt C. Voigt C. (2014). Dynamic contact mechanics on the tibial plateau of the human knee during activities of daily living. J. Biomech. 47, 2006–2012. 10.1016/j.jbiomech.2013.11.003 24296275 PMC4024103

[B21] Guess T. M. Razu S. Jahandar H. Stylianou A. (2016). Predicted loading on the menisci during gait: The effect of horn laxity. J. Biomech. 48, 1490–1498. 10.1016/j.jbiomech.2015.01.047 PMC4442067 25814179

[B22] Halonen K. S. Dzialo C. M. Mannisi M. Venäläinen M. S. de Zee M. Andersen M. S. (2017). Workflow assessing the effect of gait alterations on stresses in the medial tibial cartilage - combined musculoskeletal modelling and finite element analysis. Sci. Rep. 7, 17396. 10.1038/s41598-017-17228-x 29234021 PMC5727195

[B23] Hase K. Yamazaki N. (2002). Computer simulation study of human locomotion with a three-dimensional entire-body neuro-musculo-skeletal model (I. Acquisition of normal walking). JSME Int. J. Ser. C 45, 1040–1050. 10.1299/jsmec.45.1040

[B24] Hauch K. N. Oyen M. L. Odegard G. M. Donahue T. L. H. (2009). Nanoindentation of the insertional zones of human meniscal attachments into underlying bone. J. Mech. Behav. Biomed. Mat. 2, 339–347. 10.1016/j.jmbbm.2008.10.005 PMC2702768 19627840

[B25] Henning C. E. Clark J. R. Lynch M. A. Stallbaumer R. Yearout K. M. Vequist S. W. (1988). Arthroscopic meniscus repair with a posterior incision. Instr. Course Lect. 37, 209–221. 3047246

[B26] Howell R. Kumar N. S. Patel N. Tom J. (2014). Degenerative meniscus: Pathogenesis, diagnosis, and treatment options. World J. Orthop. 5, 597–602. 10.5312/wjo.v5.i5.597 25405088 PMC4133467

[B27] Hu J. Xin H. Chen Z. Zhang Q. Peng Y. Jin Z. (2019). The role of menisci in knee contact mechanics and secondary kinematics during human walking. Clin. Biomech. (Bristol, Avon. 61, 58–63. 10.1016/j.clinbiomech.2018.11.009 30481677

[B28] Hume D. R. Navacchia A. Rullkoetter P. J. Shelburne K. B. (2019). A lower extremity model for muscle-driven simulation of activity using explicit finite element modeling. J. Biomech. 84, 153–160. 10.1016/j.jbiomech.2018.12.040 30630624 PMC6361714

[B29] Hwang S. H. Jung K. A. Lee W. J. Yang K. H. Lee D. W. Carter A. (2012). Morphological changes of the lateral meniscus in end-stage lateral compartment osteoarthritis of the knee. Osteoarthr. Cartil. 20, 110–116. 10.1016/j.joca.2011.11.005 22133800

[B30] Kedgley A. E. Saw T. Segal N. A. Hansen U. N. Bull A. M. J. Masouros S. D. (2019). Predicting meniscal tear stability across knee-joint flexion using finite-element analysis. Knee Surg. Sports Traumatol. Arthrosc. 27, 206–214. 10.1007/s00167-018-5090-4 30097687 PMC6510819

[B66] Kempson G. E. Spivey C. J. Swanson S. A. V. Freeman M. A. R. (1971). Patterns of cartilage stiffness on normal and degenerate human femoral heads. J. Biomech. 4, 597–608. 10.1016/0021-9290(71)90049-2 5162581

[B31] Klets O. Mononen M. E. Tanska P. Nieminen M. T. Korhonen R. K. Saarakkala S. (2016). Comparison of different material models of articular cartilage in 3D computational modeling of the knee: Data from the Osteoarthritis Initiative (OAI). J. Biomech. 16, 3891–3900. 10.1016/j.jbiomech.2016.10.025 PMC5653924 27825602

[B32] Kłodowski A. Mononen M. E. Kulmala J. P. Valkeapää A. Korhonen Rk Avela J. (2016). Merge of motion analysis, multibody dynamics and finite element method for the subject-specific analysis of cartilage loading patterns during gait: Differences between rotation and moment-driven models of human knee joint. Multibody Syst. Dyn. 37, 271–290. 10.1007/s11044-015-9470-y

[B33] Kohn D. Wirth C. J. Plitz G. R. W. Maschek H. Erhardt W. Wülker N. (1992). Medial meniscus replacement by a tendon autograft. experiments in sheep. J. Bone Jt. Surg. Br. volume 74, 910–917. 10.1302/0301-620X.74B6.1447257 1447257

[B34] Kozanek M. Hosseini A. Liu F. Van de Velde S. K. Gill T. J. Rubash H. E. (2009). Tibiofemoral kinematics and condylar motion during the stance phase of gait. J. Biomech. 43, 1877–1884. 10.1016/j.jbiomech.2009.05.003 PMC2725209 19497573

[B35] Kumar D. Manal K. T. Rudolph K. S. (2013). Knee joint loading during gait in healthy controls and individuals with knee osteoarthritis. Osteoarthr. Cartil. 21, 298–305. 10.1016/j.joca.2012.11.008 PMC3804122 23182814

[B36] Kurosawa H. Fukubayashi T. Nakajima H. (1980). Load-bearing mode of the knee joint physical behavior of the knee joint with or without menisci. Clin. Orthop. Relat. Res. 149, 283–290. 10.1097/00003086-198006000-00039 7408313

[B37] Kutzner I. Heinlein B. Graichen F. Bender A. Rohlmann A. Halder A. (2010). Loading of the knee joint during activities of daily living measured *in vivo* in five subjects. J. Biomech. 43, 2164–2173. 10.1016/j.jbiomech.2010.03.046 20537336

[B38] Li G. Lopez O. Rubash H. (2001). Variability of a three-dimensional finite element model constructed using magnetic resonance images of a knee for joint contact stress analysis. J. Biomech. Eng. 123, 341–346. 10.1115/1.1385841 11563759

[B39] Li L. Yang L. Zhang K. Zhu L. Wang X. Jiang Q. (2020). Three-dimensional finite-element analysis of aggravating medial meniscus tears on knee osteoarthritis. J. Orthop. Transl. 20, 47–55. 10.1016/j.jot.2019.06.007 PMC6939112 31908933

[B40] Li L. Yang X. Yang L. Zhang K. Shi J. Zhu L. (2019). Biomechanical analysis of the effect of medial meniscus degenerative and traumatic lesions on the knee joint. Am. J. Transl. Res. 11, 542–556. eCollection 2019. 30899361 PMC6413253

[B41] McCann L. Ingham E. Jin Z. Fisher J. (2009). Influence of the meniscus on friction and degradation of cartilage in the natural knee joint. Osteoarthr. Cartil. 17, 995–1000. 10.1016/j.joca.2009.02.012 19328878

[B42] Mononen M. E. Jurvelin J. S. Korhonen R. K. (2013). Effects of radial tears and partial meniscectomy of lateral meniscus on the knee joint mechanics during the stance phase of the gait cycle—A 3D finite element study. J. Orthop. Res. 31, 1208–1217. 10.1002/jor.22358 23572353

[B43] Navacchia A. Hume D. R. Rullkoetter P. J. Shelburne K. B. (2019). A computationally efficient strategy to estimate muscle forces in a finite element musculoskeletal model of the lower limb. J. Biomech. 84, 94–102. 10.1016/j.jbiomech.2018.12.020 30616983 PMC8230727

[B44] Park S. Lee S. Yoon J. Chae S. (2019). Finite element analysis of knee and ankle joint during gait based on motion analysis. Med. Eng. Phys. 63, 33–41. 10.1016/j.medengphy.2018.11.003 30482441

[B45] Rao A. J. Erickson B. J. Cvetanovich G. L. Yanke A. B. Cole B. J. (2015). The meniscus-deficient knee. Orthop. J. Sports Med. 3, 232596711561138. 10.1177/2325967115611386 PMC4714576 26779547

[B46] Richards D. P. Barber F. A. Herbert M. A. (2008). Meniscal tear biomechanics: Loads across meniscal tears in human cadaveric knees. Orthopedics 31, 347–350. 10.3928/01477447-20080401-32 18453170

[B47] Richards R. E. Andersen M. S. Harlaar J. van den Noort J. C. (2018). Relationship between knee joint contact forces and external knee joint moments in patients with medial knee osteoarthritis: Effects of gait modifications. Osteoarthr. Cartil. 26, 1203–1214. 10.1016/j.joca.2018.04.011 29715509

[B48] Roemer F. W. Kwoh C. K. Hannon M. J. Hunter D. J. Eckstein F. Grago J. (2016). Partial meniscectomy is associated with increased risk of incident radiographic osteoarthritis and worsening cartilage damage in the following year. Eur. Radiol. 27, 404–413. 10.1007/s00330-016-4361-z 27121931 PMC5083232

[B49] Scotti C. Hirschmann M. T. Antinolfi P. Martin I. Peretti G. M. (2013). Meniscus repair and regeneration: Review on current methods and research potential. Eur. Cell. Mat. 26, 150–170. 10.22203/ecm.v026a11 24057873

[B50] Shu L. Yamamoto K. Yoshizaki R. Yao J. Takashi S. Sugita N. (2022). Multiscale finite element musculoskeletal model for intact knee dynamics. Comput. Biol. Med. 141, 105023. 10.1016/j.compbiomed.2021.105023 34772508

[B51] Sukopp M. Schall F. Hacker S. P. Ignatius A. Dürselen L. Seitz A. M. (2021). Influence of menisci on tibiofemoral contact mechanics in human knees: A systematic review. Front. Bioeng. Biotechnol. 9, 765596. 10.3389/fbioe.2021.765596 34926419 PMC8681859

[B52] Terzidis L. P. Christodoulou A. Ploumis A. Givissis P. Natsis K. Koimtzis M. (2006). Meniscal tear characteristics in young athletes with a stable knee: Arthroscopic evaluation. Am. J. Sports Med. 34, 1170–1175. 10.1177/0363546506287939 16685089

[B53] Thompson W. O. Thaete F. L. Fu F. H. Dye S. F. (1991). Tibial meniscal dynamics using three-dimensional reconstruction of magnetic resonance images. Am. J. Sports Med. 19, 210–216. 10.1177/036354659101900302 1867329

[B54] Vedi V. Williams A. Tennant S. J. Spouse E. Hunt D. M. Gedroyc W. M. (1999). Meniscal movement. An *in-vivo* study using dynamic MRI. J. Bone Jt. Surg. Br. volume 81, 37–41. 10.1302/0301-620x.81b1.0810037 10067999

[B55] Walker P. S. Arno S. Bell C. Salvadore G. Borukhov I. Oh C. (2015). Function of the medial meniscus in force transmission and stability. J. Biomech. 48, 1383–1388. 10.1016/j.jbiomech.2015.02.055 25888013

[B56] Wang S. Cai Y. Hase K. Uchida K. Kondo D. Saitou T. (2021). Estimation of knee joint angle during gait cycle using inertial measurement unit sensors: A method of sensor-to-clinical bone calibration on the lower limb skeletal model. J. Biomech. Sci. Eng. 17, 21-00196–00196. 10.1299/jbse.21-00196

[B57] Wang S. Hase K. Ota S. (2022). A computationally efficient lower limb finite element musculoskeletal framework directly driven solely by inertial measurement unit sensors. J. Biomech. Eng. 144, 051011. 10.1115/1.4053211 34897395

[B58] Wang S. Hase K. Ota S. (2021). Development of a lower limb finite element musculoskeletal gait simulation framework driven solely by inertial measurement unit sensors. Biomechanics 1, 293–306. 10.3390/biomechanics1030025 34897395

[B59] Wiess C. B. Lundberg M. Hamberg P. DeHaven K. E. Gillquist J. (1989). Non-operative treatment of meniscal tears. J. Bone Jt. Surg. 71, 811–822. 10.2106/00004623-198971060-00003 2745476

[B60] Yao J. Snibbe J. Maloney M. Lerner A. L. (2006). Stresses and strains in the medial meniscus of an ACL deficient knee under anterior loading: A finite element analysis with image-based experimental validation. J. Biomech. Eng. 128, 135–141. 10.1115/1.2132373 16532627

[B61] Yu C. Walker P. S. Dewar M. E. (2001). The effect of design variables of condylar total knees on the joint forces in step climbing based on a computer model. J. Biomech. 34, 1011–1021. 10.1016/s0021-9290(01)00060-4 11448693

[B62] Zajac F. E. (1989). Muscle and tendon: Properties, models, scaling, and application to biomechanics and motor control. Crit. Rev. Biomed. Eng. 17, 359–411. 2676342

[B63] Zhang A. L. Miller S. L. Coughlin D. G. Lotz J. C. Feeley B. T. (2015). Tibiofemoral contact pressures in radial tears of the meniscus treated with all-inside repair, inside-out repair and partial meniscectomy. Knee 22, 400–404. 10.1016/j.knee.2015.05.008 26081591

[B64] Zhang K. Li L. Yang L. Shi J. Zhu L. Liang H. (2019). Effect of degenerative and radial tears of the meniscus and resultant meniscectomy on the knee joint: A finite element analysis. J. Orthop. Transl. 18, 20–31. 10.1016/j.jot.2018.12.004 PMC6718922 31508304

[B65] Zhang K. Li L. Yang L. Shi J. Zhu L. Liang H. (2019). The biomechanical changes of load distribution with longitudinal tears of meniscal horns on knee joint: A finite element analysis. J. Orthop. Surg. Res. 14, 237. 10.1186/s13018-019-1255-1 31345248 PMC6659249

